# HMGA1 Reprograms Somatic Cells into Pluripotent Stem Cells by Inducing Stem Cell Transcriptional Networks

**DOI:** 10.1371/journal.pone.0048533

**Published:** 2012-11-15

**Authors:** Sandeep N. Shah, Candace Kerr, Leslie Cope, Elias Zambidis, Cyndi Liu, Joelle Hillion, Amy Belton, David L. Huso, Linda M. S. Resar

**Affiliations:** 1 Hematology Division, the Johns Hopkins University School of Medicine, Baltimore, Maryland, United States of America; 2 Department of Medicine, the Johns Hopkins University School of Medicine, Baltimore, Maryland, United States of America; 3 Obstetrics & Gynecology, the Johns Hopkins University School of Medicine, Baltimore, Maryland, United States of America; 4 Oncology, the Johns Hopkins University School of Medicine, Baltimore, Maryland, United States of America; 5 Biostatistics, the Johns Hopkins University School of Medicine, Baltimore, Maryland, United States of America; 6 Comparative Molecular & Pathobiology, the Johns Hopkins University School of Medicine, Baltimore, Maryland, United States of America; 7 Pathology, the Johns Hopkins University School of Medicine, Baltimore, Maryland, United States of America; 8 Pediatrics, the Johns Hopkins University School of Medicine, Baltimore, Maryland, United States of America; University of Tampere, Finland

## Abstract

**Background:**

Although recent studies have identified genes expressed in human embryonic stem cells (hESCs) that induce pluripotency, the molecular underpinnings of normal stem cell function remain poorly understood. The *high mobility group A1* (*HMGA1*) gene is highly expressed in hESCs and poorly differentiated, stem-like cancers; however, its role in these settings has been unclear.

**Methods/Principal Findings:**

We show that *HMGA1* is highly expressed in fully reprogrammed iPSCs and hESCs, with intermediate levels in ECCs and low levels in fibroblasts. When hESCs are induced to differentiate, *HMGA1* decreases and parallels that of other pluripotency factors. Conversely, forced expression of *HMGA1* blocks differentiation of hESCs. We also discovered that *HMGA1* enhances cellular reprogramming of somatic cells to iPSCs together with the Yamanaka factors (OCT4, SOX2, KLF4, cMYC – OSKM). HMGA1 increases the number and size of iPSC colonies compared to OSKM controls. Surprisingly, there was normal differentiation *in vitro* and benign teratoma formation *in vivo* of the HMGA1-derived iPSCs. During the reprogramming process, *HMGA1* induces the expression of pluripotency genes, including *SOX2*, *LIN28*, and *cMYC,* while knockdown of *HMGA1* in hESCs results in the repression of these genes. Chromatin immunoprecipitation shows that HMGA1 binds to the promoters of these pluripotency genes *in vivo*. In addition, interfering with HMGA1 function using a short hairpin RNA or a dominant-negative construct blocks cellular reprogramming to a pluripotent state.

**Conclusions:**

Our findings demonstrate for the first time that HMGA1 enhances cellular reprogramming from a somatic cell to a fully pluripotent stem cell. These findings identify a novel role for *HMGA1* as a key regulator of the stem cell state by inducing transcriptional networks that drive pluripotency. Although further studies are needed, these HMGA1 pathways could be exploited in regenerative medicine or as novel therapeutic targets for poorly differentiated, stem-like cancers.

## Introduction

Recent studies have made great strides in discovering a handful of factors important in human embryonic stem cells (hESCs) [1]–[8]. These genes (or pluripotency factors) have been used to “reprogram” normal, adult somatic cells into hESC-like cells, called induced pluripotent stem cells or iPSCs. iPSCs hold enormous promise because they could provide a source of unlimited, patient-specific stem cells for use in regenerative medicine, drug screening, or as disease models. Unfortunately, the derivation of iPSCs is inefficient, and the ability to maintain and differentiate iPSCs remains a technical hurdle in the field. Moreover, iPSCs, and even normal hESCs, can acquire abnormal karyotypes and invasive properties, recapitulating features of cancer cells [9]–[13]. Thus, a better understanding of the molecular mechanisms responsible for normal stem cell properties in hESCs and iPSCs is needed before these cells can be safely used in the clinic. Studies to elucidate the underpinnings of normal hESCs and fully reprogrammed iPSCs should also provide insight relevant to cancer because pluripotent stem cells and cancer cells share a subset of transcriptional networks and properties [9]. It will be critical, however, to identify the molecular mechanisms that distinguish normal stem cells from malignantly transformed, stem-like cells.

The high *mobility group A1* (*HMGA1*) gene is highly expressed during embryogenesis and enriched in hESCs [9], hematopoietic stem cells (HSCs) [13]–[16], and poorly differentiated or refractory cancers [9], [15]–[37], with low or undetectable expression in adult, differentiated tissues. This gene encodes the HMGA1a and HMGA1b protein isoforms [38]–[39], which are members of the HMGA superfamily of chromatin remodeling proteins that include HMGA1a, HMGA1b, and HMGA2 [38]–[43]. HMGA proteins are low molecular weight (thus high mobility group) proteins that bind to AT-rich regions in chromatin and orchestrate the assembly of transcription factor complexes to modulate chromatin structure and regulate gene expression [27], [29], [30], [32], [34]–[36], [42]–[45]. HMGA proteins induce malignant transformation in cultured cells and cause aggressive tumors in transgenic mice [18]–[19], [21]–[37], [40]–[46]. The tumors from HMGA1 mice can be serially transplanted, indicating that they have the stem cell property of long term self-renewal [32]. *HMGA1* expression is highest in cultured cells that are derived from poorly differentiated cancers, including breast [21], [45], prostate [23], pancreatic [31], uterine [26], colon [34], and lung [30] cancers as compared to cell lines from more differentiated tumors. Expression of *HMGA1* is also associated with poor differentiation status in solid tumors arising from different tissues and embryonic origins [9], [26], [30], [34], [47]–[49]. Moreover, *HMGA1* overexpression portends a poor outcome in diverse tumors, including cancers of the pancreas [31], brain [9], [48], bladder [9], lung [49], and breast [9], [47]. *HMGA1* is also enriched in refractory hematopoietic cancers [15]–[16], [18]–[19], [29], [33] and in human iPSCs [13]. Together, these studies in cancer and pluripotent stem cells suggest that HMGA1 could function to reprogram cells to a more primitive, undifferentiated, stem-like state.

Previous studies in cancer cells have demonstrated that HMGA1 directly activates specific genes involved in tumor growth and progression, including proliferation, migration, invasion, angiogenesis, genetic instability, resistance to cell death, immune evasion, and an epithelial-mesenchymal transition in cancer cells, although its role in embryonic stem cells is poorly understood [23,26–30,32–36.,45]. Here, we report that HMGA1 promotes the cellular reprogramming of adult somatic cells to undifferentiated, fully pluripotent stem cells (iPSCs). We also identify transcriptional networks induced by *HMGA1* to drive the stem cell phenotype in pluripotent stem cells. Our studies provide new insights into the role of HMGA1 in development, stem cells, and cellular reprogramming.

## Results

### *HMGA1* Expression Decreases with Differentiation in hESCs

**Figure 1:**
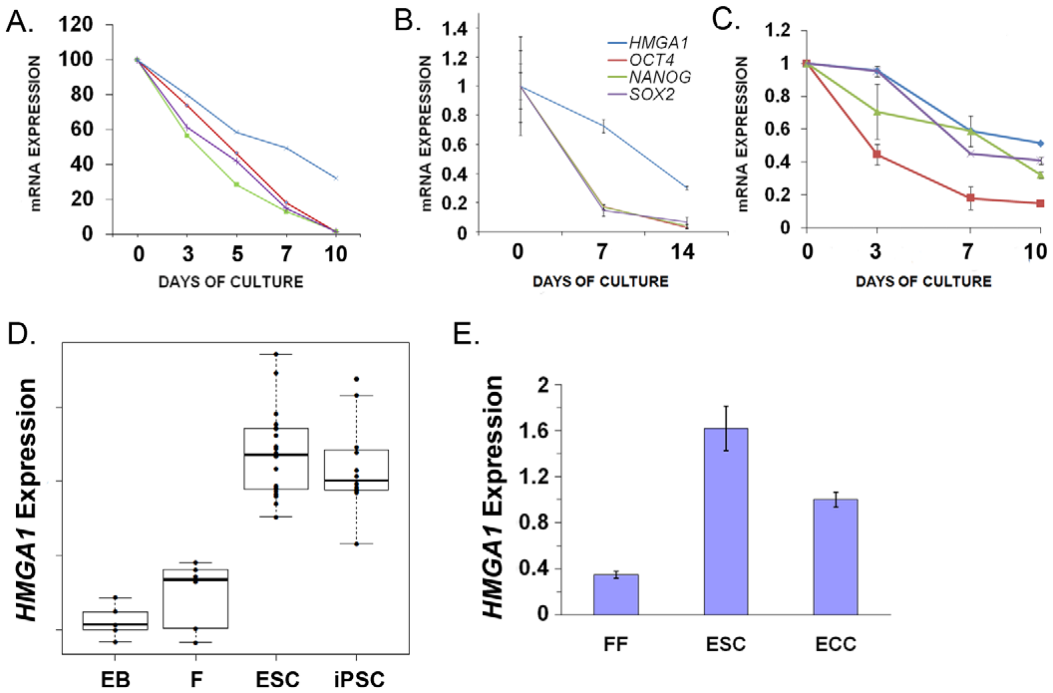
*HMGA1* expression falls with differentiation in hESCs and parallels that of other pluripotency genes. **A**) H1 hESCs were cultured under conditions to promote hematopoietic differentiation. By days 7–10, hESCs differentiate into mesodermal-hematoendothelial (MHE) colonies and fully differentiated progeny of all hematopoietic lineages. (See ref. 50 and GEO accession number GSE12531 for microarray data). **B**) *HMGA1* expression falls in hESCs cultured under conditions to promote neuroectodermal differentiation. *HMGA1* decreases with *OCT4*, *NANOG*, and *SOX2* by day 10, as shown by qRT-PCR. **C**) Similarly, *HMGA1* expression falls when hESCs differentiate into mesoderm as shown by qRT-PCR. **D**) Expression levels of *HMGA1* in embryoid bodies (B), fibroblasts (F), hESCs (H), and iPSCs (I) from a published database [8]. **E**) *HMGA1* expression in fetal fibroblasts, H9 hESCs, and embryonal carcinoma cells (ECC) was assessed by qRT-PCR; *HMGA1* expression in the H9 hESCs was arbitrarily assigned a value of 1.0.

To better define the role of *HMGA1* in pluripotent stem cells, we investigated its expression in hESCs during differentiation. First, we assessed *HMGA1* expression patterns in H1 hESCs induced to differentiate into blood cells in an established model of hematopoiesis [50]. *HMGA1* mRNA was highest at day 0, with levels dropping dramatically as the hematopoietic cells differentiate (day 10; Fig. 1A) by microarray gene expression profile analysis (microarray data found in Gene Expression Omnibus, accession number GSE12531). Notably, the levels of *HMGA1* closely parallel those of the embryonic stem cell and pluripotency factors *NANOG, OCT4,* and *SOX2.* These results were confirmed by quantitative RT-PCR (qRT-PCR; data not shown). When hESCs are forced to differentiate into neuroectodermal lineages, we also found that *HMGA1* expression decreases by qRT-PCR, similar to *NANOG, OCT4,* and *SOX2* (Fig. 1B). Likewise, *HMGA1* expression falls and mirrors that of *NANOG, OCT4,* and *SOX2* during mesodermal differentiation, as demonstrated by qRT-PCR (Fig. 1C). To further investigate the role of *HMGA1* in pluripotency, we compared *HMGA1* expression in embryoid bodies, fibroblasts, hESCs, and iPSCs from a study of global gene expression profile analyses [8]. We found that *HMGA* mRNA levels were highest in the pluripotent hESCs and iPSCs with lower levels in differentiated cells (embryoid bodies and fibroblasts; Fig. 1D). Using qRT-PCR, we found that cultured cancer cells derived from a germ cell tumor (Tera-2 embryonal cancer cell or ECC line) have ∼50% lower *HMGA1* mRNA levels compared to hESCs (Fig. 1E). These findings indicate that *HMGA1* expression is similar to that of key pluripotency factors as hESCs differentiate and suggest that *HMGA1* could function in maintaining an undifferentiated state in normal hESCs.

### *HMGA1* Blocks Differentiation in hESCs

**Figure 2:**
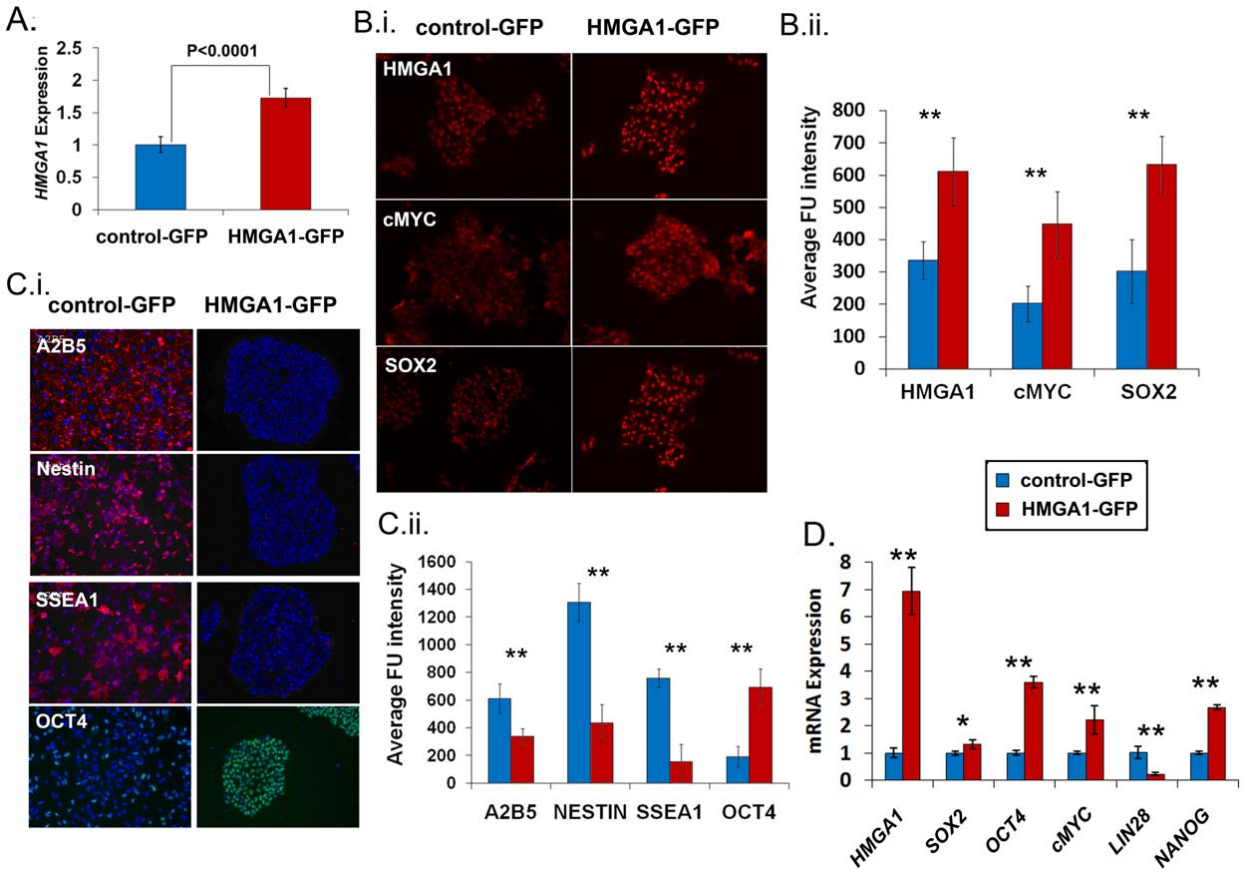
HMGA1 drives a de-differentiated state. **A**) *HMGA1* expression is increased in hESCs 3 days after transduction with the HMGA1-GFP lentivirus (HMGA1-GFP) compared to control hESCs (control-GFP). Bars, mean ± standard deviation. **B**) **i.** HMGA1, cMYC and SOX2 proteins are up-regulated in HMGA1-GFP (red) compared to control-GFP (blue) hESCs. **ii.** Fluorescence was assessed quantitatively using MetaMorph (Universal Imaging) version 7.7 (p<0.001 for all genes in HMGA1-GFP compared to control-GFP hESCs). **C**) **i.** Control-GFP hESCs (blue) differentiate after treatment with neuroectodermal differentiation factors and express neural markers (left three panels. red: A2B5-top, Nestin–middle, SSEA1-lower panel), while the HMGA1-GFP hESCs (red) remain embryonic stem cell colonies and express hESC markers (OCT4), but no neural markers. DAPI was used to stain nuclei. **ii.** Quantitative analysis of HMGA1 and the stem cell markers (cMYC and SOX-2) are shown; (p<0.001 for all proteins assessed in HMGA1-GFP compared to control-GFP hESCs). **D**) The pluripotency genes *SOX2, OCT4*, *cMYC*, and *NANOG* are up-regulated in the HMGA1-GFP hESCs (red) compared to controls (blue) in hESCs cultured in conditions that promote neuroectodermal differentiation. Levels of exogenous HMGA1 are also shown.

To further investigate the role of HMGA1 in the maintenance of an undifferentiated state, we determined if forced expression of *HMGA1* in hESCs will affect differentiation. We therefore engineered H9 hESCs to express *HMGA1* by transducing these cells with a lentiviral vector expressing *HMGA1* linked to green fluorescent protein (GFP) [26]. Control cells were transduced with a lentiviral vector expressing GFP alone [26]. The hESCs transduced with the HMGA1a lentivector showed a corresponding increase in *HMGA1* mRNA levels compared to the control (Fig. 2A). By immunofluorescent cytochemistry, we documented that the HMGA1 protein was increased in the H9 hESCs transduced with the *HMGA1* lentivirus compared to the control lentivirus (Fig. 2B, upper panels). In addition, we found that both SOX2 and cMYC proteins were also increased in HMGA1 hESCs compared to controls (Fig. 2B). To better define the role of HMGA1 in stem cells, the transduced HMGA1 and control hESCs were cultured under conditions to promote differentiation into neuroectoderm as described previously [51]–[52]. Strikingly, hESCs expressing exogenous *HMGA1* showed no evidence for differentiation into neuroectoderm. The HMGA1-expressing cells maintained normal hESC morphology and expression of pluripotency markers (Fig. 2C–D); there was also no expression of differentiation markers (Fig. 2C). In contrast, the control-GFP cells underwent dramatic morphological changes, growing as a monolayer. Likewise, control cells expressed neuronal markers (A2B5, Nestin, and SSEA1) after culture in differentiation conditions (Fig. 2C). By qRT-PCR, the *HMGA1* cells expressed significantly higher levels of pluripotency genes after 7 days in differentiation conditions, including *OCT4* (p<0.01), *SOX2* (p<0.05), *cMYC* (p<0.01), and *NANOG* (p<0.01) compared to the controls (Fig. 2D). In addition, exogenous *HMGA1* levels remained high (7-fold) compared to controls. Surprisingly, *LIN28* expression was not increased in the HMGA1-GFP cells, indicating that HMGA1 blocks differentiation without maintaining high levels of *LIN28*. In this setting, the differentiating factors could be the primary regulators of *LIN28*. To determine if HMGA1 alters growth rates in hESCs, we also performed proliferation (MTT) assays and found no significant difference in growth rates (Fig. S1). These results indicate that constitutive expression of *HMGA1* blocks differentiation and maintains hESCs in an undifferentiated, stem-like state.

### HMGA1 Enhances Cellular Reprogramming to Fully Pluripotent iPSCs

**Figure 3:**
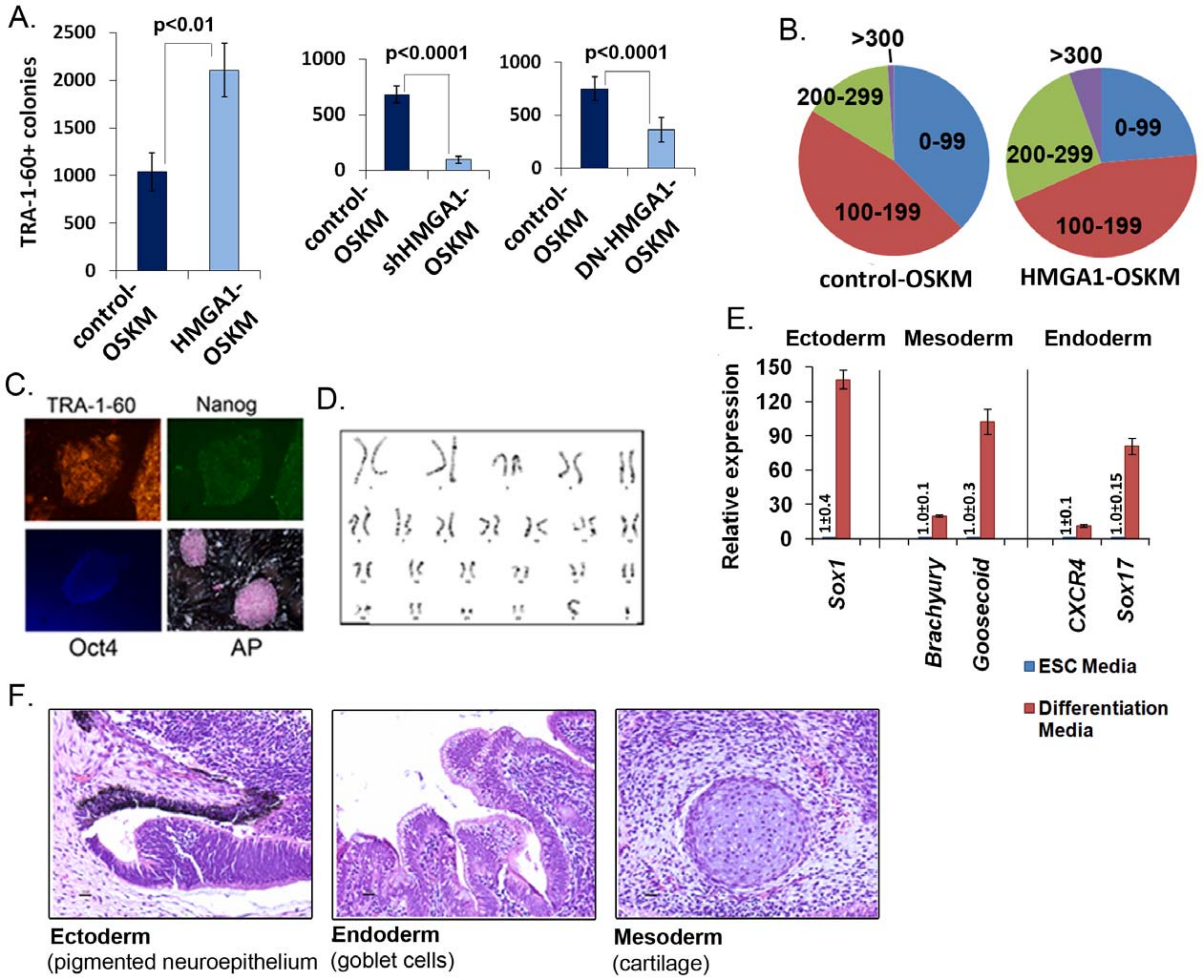
*HMGA1* promotes cellular reprogramming of MSCs to fully pluripotent iPSCs. **A**) Reprogramming with HMGA1-OSKM results in more TRA-1-60+iPSC colonies compared to controls, while shHMGA1 and dominant-negative HMGA1 (DN-HMGA1) decreases the number of colonies, as assayed on day 16 following retroviral transduction. **B**) The HMGA1-OSKM TRA-1-60+colonies are significantly larger than the control-OSKM colonies; sizes: µm. **C**) The HMGA1-OSKM colonies stain positively for standard stem cell markers (TRA-1-60, Nanog, Oct4, and Alkaline Phosphatase or AP). **D**) Established HMGA1-OSKM colonies have a normal karyotype. **E**) HMGA1-OSKM colonies express representative markers from 3 germ layers when induced to differentiate *in vitro.* HMGA1-OSKM colonies were either cultured in standard hESC media or under conditions for differentiation into ectoderm, mesoderm, or endoderm. The appropriate genes for each condition were expressed. **F**) HMGA1-OSKM colonies form benign teratomas with constituents from all three germ layers.

Based on the above findings, we hypothesized that *HMGA1* promotes cellular reprogramming and could enhance the derivation of iPSCs. To test this hypothesis, we used standard retroviral reprogramming technology to transduce bone-marrow derived, commercial, adult mesenchymal stem cells (MSCs). MSCs (100,000 cells per reprogramming experiment) were transduced with the four Yamanaka factors (OCT2, SOX4, KLF4, and cMYC or OSKM [1]) plus HMGA1 or control, all expressed by pMX retroviral vectors as previously described [53]–[54]. The addition of HMGA1 to OSKM (denoted HMGA1-OSKM) resulted in a consistent 2-fold increase in TRA-1-60+iPSC colonies compared to the control-OSKM transduction (Fig. 3A). Previous studies showed that TRA-1-60+staining is a reliable early indicator of fully reprogrammed iPSC colonies [10]–[11], [54]–[55]. Moreover, we found that the early HMGA1-OSKM TRA-1-60+colonies were significantly larger than their control-OSKM counterparts (Fig. 3B), indicating that HMGA1 enhances the reprogramming rate, stem cell survival, proliferation, or a combination of these factors during iPSC generation. To determine if HMGA1 is required during cellular reprogramming to iPSCs, we blocked *HMGA1* expression or function using a short hairpin RNA (shRNA) to *HMGA1* or dominant-negative construct, respectively. MSCs were transduced with OSKM as described above. Forty-eight hours after reprogramming with OSKM, cells were transduced with a lentivirus containing *HMGA1* shRNA or control shRNA. Strikingly, we found that there was a marked decrease in TRA-1-60+colonies in the cells treated with *HMGA1* shRNA as compared to controls (p<0.0001; Fig. 3A). Similarly, we found that the dominant-negative HMGA1 also blocked cellular reprogramming to iPSCs (p<0.0001; Fig. 3A).

To determine if the MSC-derived TRA-1-60+colonies are fully reprogrammed and express other standard stem cell markers after transduction with HMGA1-OSKM, we selected and subcultured colonies for further analysis with immunoflourescent intracellular staining after 6 passages. As expected for hESCs and fully reprogrammed iPSCs, the HMGA1-OSKM clones also expressed OCT4, NANOG, and alkaline phosphatase (AP) (Fig. 3C). The HMGA1-OSKM clone (HMGA1-OSKM-4) had a normal karyotype after culturing the cells for>10 passages (Fig. 3D). In addition, the HMGA1-OSKM iPSCs could be fully differentiated into neuronal or meso/endoderm lineages *in vitro* (Fig. 3E) and generate teratomas with all three germ layers represented (Fig. 3F). There was no detectable expression of exogenous OSKM from the retroviral vectors in the HMGA1-OSKM clone 57 days following transduction, although the vectors were detectable 21 days after transduction (data not shown).

Next, we investigated whether HMGA1 enhances cellular reprogramming of other somatic cells. We therefore transduced fetal lung fibroblasts (IMR90) with the HMGA1-OSKM or control-OSKM retroviruses. Similar to our data in MSCs, we found that HMGA1 significantly enhances the size and number of TRA-1-60+colonies (Fig. S2). We observed an increase in the number of iPSC colonies by about 2-fold with HMGA1 in the reprogramming cocktail. Taken together, our results demonstrate that HMGA1 promotes cellular reprogramming to an undifferentiated, pluripotent stem-like state in somatic cells of different origins (MSCs, IMR90 fetal lung fibroblasts).

### HMGA1 Modulates the Expression of Pluripotency Genes

**Figure 4:**
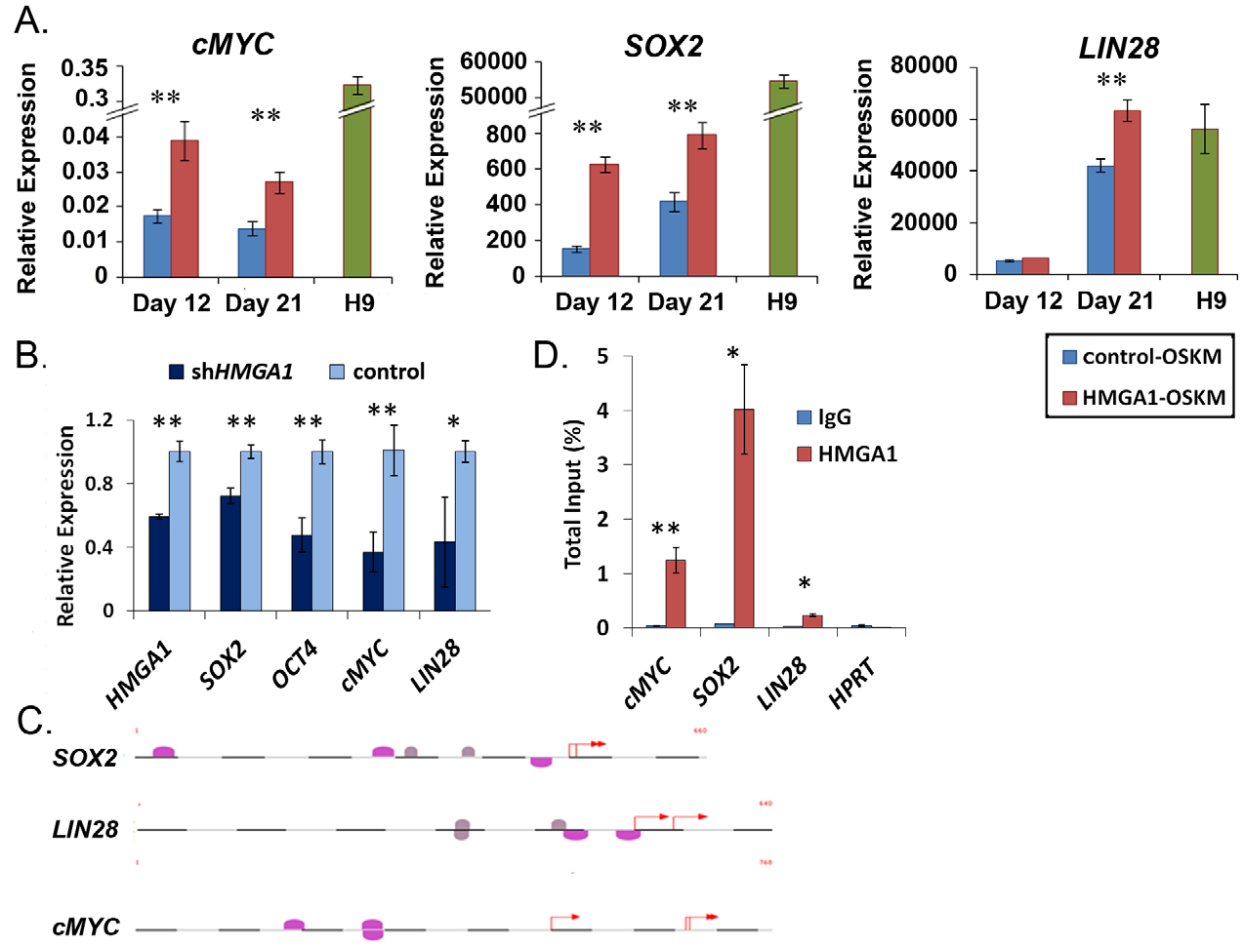
HMGA1 regulates pluripotency genes. **A**) Endogenous *SOX2*, *LIN28*, and *cMYC* are induced more in MSCs reprogrammed with HMGA1-OSKM (red) compared to control pools (blue). Gene expression in H9 hESCs is shown as a reference (green). **B**) Pluripotency genes (*SOX2, OCT4, cMYC, LIN28*) are repressed following shRNA-mediated knockdown of *HMGA1.* *p<0.01, **p<0.00001. **C**) The promoter regions of pluripotency genes contain putative HMGA1 DNA binding sites (pink ovals) located near putative NFκB sites (grey circles). **D**) Chromatin immunoprecipitation in hESCs shows enrichment in HMGA binding in the promoters of *SOX2, cMYC* and *LIN28.* IgG was used as a negative control; cMYC antibody and primers to the *B23* promoter were used as a positive control. *p<0.01, **p<0.001.

Because HMGA1 functions by modulating gene expression, we hypothesized that it promotes pluripotency by inducing stem cell transcriptional networks. We therefore assessed the expression of a subset of endogenous, human embryonic stem cell/pluripotency genes (*OCT4*, *SOX2*, *cMYC*, *NANOG*, *LIN28*, *REX1*, *hTERT*) at early stages (day 12 and 21) in the reprogramming pools following transduction of MSCs with HMGA1-OSKM or control-OSKM. At day 12, expression of both *SOX2* (p<0.001) and *cMYC* (p<0.01) were 2–3-fold higher in the HMGA1-OSKM pools compared to the control-OSKM cells (Fig. 4A). At day 21, expression of *SOX2* (p<0.01), *LIN28* (p<0.01), and *cMYC* (p<0.01) were all significantly increased in the MSCs reprogrammed with HMGA1-OSKM compared to the control-OSKM pools (Fig. 4A). Surprisingly, the expression of the other stem cell genes was not significantly altered in the HMGA1-OSKM pools compared to controls (data not shown). There were no significant changes in expression of the exogenous, murine *OCT4*, *SOX2*, *KLF4*, or *cMYC* delivered by retrovirus (see Fig. S3 for qRT-PCR results and Table S1 for primers specific to the murine OSKM delivered by retrovirus). Of note, the established iPSC clones (after 10 passages) generated by transduction of HMGA1-OSKM or control-OSKM had similar expression of all pluripotency genes assessed, and the expression levels were similar to those observed in hESCs (data not shown). These results suggest that HMGA1 enhances the derivation of iPSCs by inducing the expression of a subset of pluripotency-associated genes early in the reprogramming process.

To determine if HMGA1 also modulates expression of pluripotency genes in hESCs, we knocked-down *HMGA1* expression in H9 hESCs using shRNA (sh*HMGA1*) [56] and assessed the expression of seven embryonic stem cell/pluripotency genes (*OCT4*, *SOX2*, *cMYC*, *NANOG*, *LIN28*, *REX1*, *hTERT*). We found that *SOX2*, *OCT4*, *cMYC*, and *LIN28* were all significantly repressed in the hESCs 96 hours following shRNA-mediated knock-down of *HMGA1* (Fig. 4B). These results were compared to H9 hESCs treated with a control shRNA vector. To further rule-out off-target effects of the sh*HMGA1*, we also assessed the expression of these genes after knocking down *HMGA1* using *HMGA1* siRNA (Dharmacon), which targets a different sequence in the *HMGA1* mRNA [30]. Using this approach, *SOX2*, *OCT4*, *cMYC*, and *LIN28* were also repressed after transduction with the si*HMGA1* after 24 hours (Fig. S4). Expression of the other pluripotency genes did not change significantly, although both approaches (shRNA and siRNA) resulted in significant knock-down of *HMGA1* mRNA. Of note, there were no gross changes in colony morphology or proliferation at these early time points using siRNA or shRNA. Together, our results indicate that HMGA1 modulates a specific subset of stem cell/pluripotency genes during the generation of iPSCs and in fully pluripotent hESCs.

### HMGA1 Binds to Pluripotency Gene Promoters

Because HMGA1 binds chromatin to modulate gene expression [32], we hypothesized that HMGA1 induces an undifferentiated, pluripotent state by binding to DNA and enhancing transcriptional networks downstream of the stem cell gene targets, such as *cMYC*, *SOX2*, and *LIN28* genes. Using MatInspector [57], we found that the promoter regions of *cMYC*, *SOX2*, and *LIN28* contain AT-rich regions with putative HMGA1 binding sites (Fig. 4C). To determine if HMGA1 binds to the promoters of these genes *in vivo*, we performed chromatin immunoprecipitation in H9 hESCs. We found that the promoter regions of *cMYC*, *SOX2,* and *LIN28* with the putative HMGA1 binding sites were enriched in HMGA1-binding (Fig. 4D). Furthermore, there was no demonstrable binding of HMGA1 to the negative control promoter, *HPRT*, which was shown in previous studies to lack HMGA1 binding [29]. These results indicate that HMGA1 binds directly to the *cMYC*, *SOX2,* and *LIN28* promoters and suggest that HMGA1 enhances pluripotency by inducing expression of *cMYC*, *SOX2,* and *LIN28* early in reprogramming.

### HMGA1 iPSCs have Promoter Methylation Patterns similar to hESCs

Prior studies suggest that epigenetic reprogramming is involved in the induction of pluripotent stem cells [12]. We therefore investigated promoter methylation patterns in the HMGA1 iPSCs and reprogramming pools using the Illumina Infinium Methylation27 platform, which includes probes for 27,576 loci (Fig. S5). Promoter methylation was assessed from genomic DNA isolated from a fully characterized iPSC clone (HMGA1-OSKM-A4) and MSC reprogramming pools 12 and 21 days after transduction with HMGA1-OSKM or control-OSKM. For comparison, we also included cancer cells, hESCs, fetal fibroblasts, MSCs, and previously characterized iPSCs for which promoter methylation was assessed in a previous study [12]. The promoter methylation patterns in the fully reprogrammed iPSCs generated by HMGA1–OSKM or control-OSKM were similar to that of hESCs and other iPSCs. There were no significant differences in the relative number of CpG sites with high or low levels of methylation in the HMGA1-OSKM reprogramming pools compared to the OSKM controls. These findings indicate that the iPSCs reprogrammed with HMGA1 have similar methylation patterns to hESCs and other iPSCs and suggest that the enhanced reprogramming by HMGA1 does not occur through large changes in global methylation patterns.

## Discussion

Here, we provide compelling evidence that HMGA1 plays a key role in cellular reprogramming to a pluripotent stem cell and the maintenance of the undifferentiated state: 1.) *HMGA1* expression is enriched in hESCs and fully reprogrammed iPSCs, with intermediate levels in cancer cells and lower levels in differentiated fibroblasts, 2.) HMGA1 enhances cellular reprogramming of somatic cells to a pluripotent state, while forced expression blocks differentiation in hESCs, and 3.) HMGA1 binds to promoters and induces expression of other pluripotency factors, whereas knock-down of HMGA1 represses pluripotency factors. In multiple settings (bone marrow-derived MSCs, fetal lung fibroblasts), more iPSC colonies formed when HMGA1 was added to the OSKM reprogramming cocktail, and the colony size was greater in most cases. Interestingly, mice that are null for *HMGA1* have normal early development [58], while mice deficient in the HMGA family member, *HMGA2*, have a pygmy phenotype, but otherwise normal early development [59]. Because both HMGA1 and HMGA2 proteins have a high level of homology and similar functions in experimental models [18], [45], it is possible that there is functional redundancy, and knock-out of HMGA1 is partially compensated for by HMGA2. Genetic experiments are underway to address this issue.

We also discovered that germ cell tumor cells express less *HMGA1* than fully reprogrammed iPSCs and hESCs, suggesting that a critical level of *HMGA1* may be required for a fully reprogrammed, pluripotent, stem-like phenotype in contrast to a malignant phenotype. Perhaps the addition of *HMGA1* in the reprogramming cocktail results in a greater proportion of cells that cross a critical *HMGA1* gene threshold and produce fully reprogrammed iPSCs. Prior studies have shown that reprogramming cancer cell lines with retroviral delivery of pluripotency genes results in ES-like cells with slower growth rates as well as the ability to form benign teratomas *in vivo* and respond to differentiating agents *in vitro* [60]–[63]. iPSCs derived from a colon cancer cell line (DLD1) also became more sensitive to the cytotoxic agent, 5-FU [60], although subsequent studies found that iPSCs generated from another cancer cell line (HuCC-T1 choriocarcinoma cells) became invasive and lost their response to differentiating or cytotoxic agents *in vitro* as well as their ability to form benign teratomas *in vivo* when cultured for longer time periods (>120 days following reprogramming) [63]. These cells had activation of endogenous *cMYC* when they lost their potential for pluripotency, indicating that the reprogramming was reversible. Together, these studies and our findings presented here suggest that strategies to induce expression of *HMGA1* and that of other pluripotent genes could reprogram malignant tumors into cells that respond to differentiating agents. Further studies are needed to test this hypothesis and could lead to the development of novel therapeutic strategies to reprogram cancer cells.

The enhanced reprogramming by HMGA1 could also be exploited to generate patient-specific iPSCs for disease modeling, drug testing, or regenerative medicine with patient-derived cells. Surprisingly, we did not find evidence for malignant transformation in the iPSCs reprogrammed by HMGA1-OSKM. Rather, there was an increase in the number of TRA-1-60+staining clones when HMGA1 was included in the reprogramming cocktail, which is a reliable marker for fully pluripotent iPSCs [54]. Moreover, the teratoma assay showed differentiated tissues from all three germ layers, further documenting the pluripotent state of the HMGA1-OSKM iPSCs. The iPSCs could also be differentiated into embryoid bodies, neuroectoderm, or mesoderm, indicating that the addition of HMGA1 did not interfere with the pluripotent/differentiation potential of the iPSCs. The karyotype was normal and promoter methylation patterns were similar to those observed in hESCs. In preliminary studies, we also found that HMGA1 significantly enhances reprogramming of mononuclear blood cells (MBCs) to TRA-1-60+colonies using an episomal vector approach (unpublished data). This latter approach has several advantages for potential clinical uses. Most notably, the reprogramming vectors do not integrate into the genome, thus avoiding the complication of activating (or inactivating) critical loci or otherwise disrupting the genome. Episomal vectors are also ultimately lost as the cells successively divide and the iPSCs generated from this approach may be less immunogenic [64].

To determine how HMGA1 promotes an undifferentiated, pluripotent stem cell state, we investigated the expression of stem cell transcriptional networks and found several key genes that are induced by HMGA1 in the iPSC pools early in reprogramming, including *SOX2*, *LIN28*, and *cMYC*. We also discovered that HMGA1 binds to the promoters of these genes *in vivo* in hESCs, suggesting that HMGA1 directly induces their expression. Interestingly, most HMGA1 transcriptional targets have a consensus DNA binding site for NF-κB in the promoter regions near the AT-rich site where HMGA1 binds [32], indicating that these factors could function together in activating cellular pathways in stem cells. Based on the MatInspector computational algorithm and published literature, the pluripotency genes induced by HMGA1 also include NF-κB sites near the HMGA1 binding sites, including *LIN28*, *SOX2, cMYC*, and *OCT4*, (Fig. 4C), indicating that both HMGA1 and NF-κB could promote cellular reprogramming by inducing expression of pluripotency genes. A recent study identified *HMGA1* as a gene whose translation is induced by Lin28 in hESCs [65]. This suggests that a positive feedback loop could exist whereby Lin28 enhances HMGA1 translation and HMGA1, in turn, feeds back to induce expression of *LIN28,* along with other pluripotency genes, to activate stem cell networks. Surprisingly, not all pluripotency genes with predicted HMGA1 DNA binding sites in their promoters were induced in the HMGA1 reprogramming pools, indicating that HMGA1 induces a specific stem cell signature in this setting. Further studies are needed to elucidate all of the critical networks related to HMGA1 that drive a fully pluripotent state in iPSCs and stem cells.

In summary, we demonstrate for the first time that HMGA1 enhances cellular reprogramming of somatic cells to a fully pluripotent state. We also discovered that HMGA1 promotes an undifferentiated, pluripotent state and blocks differentiation. These findings provide insight into HMGA1 function in hESCs that could be exploited for patient-derived iPSCs for use in regenerative medicine. Although additional studies are needed, our findings also suggest that HMGA1 transcriptional networks are important in reprogramming normal cells into stem-like, malignant cancer cells and that these pathways could be targeted in therapy.

## Materials and Methods

### Ethics Statement

All animal experiments were conducted in accordance with a protocol approved by the Johns Hopkins University Animal Care and Use Committee (protocol# MO11M279). All mice were housed in a sterile environment where they had free access to food and water as outlined in our institutional guidelines.

### Cell Culture

MSC1640 mesenchymal stem cells (AllCells, LLC) were maintained in DMEM (low glucose) with 10% FBS, 1× NEAA, 1× L-Glutamine, 1× Antibiotic/Antimycotic (Invitrogen) and bFGF (1 ng/ml). IMR90 fetal lung fibroblast cells (ATCC) and cultured similar to MSC1640, but without bFGF. iPSCs and hESCs (H1 and H9 from WiCell) were maintained in ES media: DMEM/F12, 20% Knockout Serum Replacement, 1× NEAA, 1× L-Glutamine, 1× Antibiotic-Antimycotic, 1 mM 2-Mercaptoethanol, bFGF (10 ng/ml for iPSCs or 8 ng/ml for hESCs). The human embryonal carcinoma line, NTERA-2 cl.D1 (ATCC) was cultured on matrigel-coated plates under conditions as previously described [66].

### Transduction and Reprogramming Vectors

The pMXs-HMGA1 vector was made by restricting the pMXs retroviral vector [48] at the BstXI site and subsequently made blunt using Klenow. The human *HMGA1a* cDNA was inserted into pMXs following amplification from pooled human RNA with the following primers: (F) 5′-AGCCAATCCTATGGACCTGCTCCTTAGAGAAGGGAA-3′; (R) 5′-AGCCAATCCTATGGAAAGCTGTCCAGTCCCAGAA-3′. The correct HMGA1 sequence construct was confirmed by sequencing. The pMXs-DN-HMGA1 vector was made by isolating HMGI (mII,III) from pcDNA3.1/zeo.HMGI(mII,mIII) (a generous gift from Raymond Reeves, Washington State University, and described in detail in [67]) by restricting with HINDIII/BamHI; blunt end cloning was used to introduce the cDNA into pMXs (as described above). The empty pMXs vector was used as a negative control.

### RNA Interference

The short-hairpin RNA interference vector for HMGA1 targets 5′-CAACTCCAGGAAGGAAACCAA-3′ and has been described elsewhere [56]. The empty vector was used as a negative control in knockdown experiments similar to a previous study [34]. The *HMGA1* siRNA targets 5′-AGCGAAGTGCCAACACCTA-3′ and was obtained from Dharmacon [26]. siCONTROL (Dharmacon), which contains 4 siRNAs without matches to human, mouse, or rat genes, was used as the negative control for the siRNA experiments.

### Retroviral Infection and Reprogramming Protocols

DNA vectors pMX-OCT4, pMX-SOX2, pMX-cMYC, and pMX-KLF4 (*M. musculus* genes) were generously provided by Linzhao Cheng (Johns Hopkins University). Retrovirus containing pMX-OCT4, pMX-SOX2, pMX-cMYC, pMX-KLF4, pMX-HMGA1, pMX-DN-HMGA1, pMX-shHMGA1, or pMX empty vector were produced and used for infection as previously described [49]. During days 7–16 following transduction, cells were maintained in ES media+0.5 mM sodium butyrate as previously reported [11].

### Immunostaining

Live staining for TRA-1-60 to identify fully reprogrammed colonies was performed as described [11] using anti-TRA-1-60 (Millipore) and a mouse secondary antibody at a dilution of 1∶200 and 1∶400, respectively, premixed in hESC media. The TRA-1-60+putative iPSC colonies were further characterized for stem cell markers after fixation with paraformaldehyde as previously described [11]. To assess stem cell markers, the putative iPSC colonies were stained for immunoflourescence with the following antibodies: TRA-1-60 (1∶200, Millipore), NANOG (1∶1000, Abcam), OCT4 (1∶100, Santa Cruz Biotechnology), SOX2 (1∶100), cMYC and HMGA1 (1∶100) followed by secondary antibodies conjugated to a Alexa Probes (Molecular Devices) as previously described [68]. Immunofluorescence intensity calculations were performed using MetaMorph (Universal Imaging) version 7.7. Alkaline phosphatase staining was performed using the Alkaline Phosphatase detection kit (Millipore).

### MTT Cell Proliferation Assays

Cells (5,000) were plated onto 96 well plates coated in matrigel with conditioned media obtained from mouse embryonic fibroblasts as described [29]. The media was replaced daily. MTT assays (Invitrogen) were performed daily for 5 days using 100 µl of MTT solution (5 mg/ml) added to each well and incubated for 3 h at 37°C according to manufacturer’s instructions. The formed MTT formazan crystals were dissolved with 500 µL DMSO, and the spectrophotometric assay was carried out at 590 nm as described. Each condition was done in quadruplicate, and 2 independent experiments were performed.

### Gene Expression Analysis with Quantitative, Reverse Transcription PCR

Total RNA was isolated using the miRNeasy kit (Qiagen) and analyzed by quantitative reverse transcription PCR (qRT-PCR) as we previously described [29]. The sequences for the forward and reverse primers are listed in Table S1. For transgene expression analysis, one primer was designed with sequence from the pMX retroviral vector and the other primer was designed with sequence from the gene of interest. The expression level of each gene was normalized to the *TATA-binding protein* (*TBP*) gene.

### Chromatin Immunoprecipitation (ChIP)

H9 hESCs cells (approximately 5 million) were washed twice with PBS and collected following incubation in trypsin (0.25%). Protein was cross-linked to DNA by treatment with formaldehyde for 8 minutes, after which the reaction was stopped with glycine. Cells were pelleted and resuspended in cell lysis buffer along with a protease inhibitor cocktail (Roche). After 10 minutes on ice, the nuclei were pelleted and resuspended in 200 µl nuclei lysis buffer with protease inhibitors. Chromatin was sheared by sonication using the BioRupter® (Diagenode) for two runs of 10 cycles. ChIP buffer was added to the sonicated samples to a final volume of 1 ml. ChIP was performed either by using the Auto-histone ChIP-seq kit on the SX-8G IP-Star® Compact platform (Diagenode) or the SimpleChIP Enzymatic Chromatin IP kit (Cell Signaling Technology) according to the manufacturers’ instructions.

The sheared DNA-protein complexes were immunoprecipitated using antibodies to HMGA1 as we described [29]. An IgG antibody was used as a negative control. Sequence from an approximately 200 base pair regions of the promoter of the pluripotency genes containing the predicted HMGA1 binding site was amplified using PCR. As a positive control, we amplified the 200 bp region containing the cMYC DNA binding site in the *B23* promoter and performed immunoprecipitation with the cMYC antibody as previously described [69].

### Genome-wide DNA Methylation Analysis

To assess global promoter methylation, we used the Infinium (Illumina, Inc.) platform to analyze bisulfate-treated DNA (EX DNA Methylation kit, Zymo Research) containing 27,578 informative sites near promoter regions as previously described [10]. Briefly, β values are generated as the signal of methylation-specific probe over the sum of the signals of the methylated and unmethylated-specific probes. The score of 1.0 is assigned for full methylation of a specific CpG site, 0 for the absence of methylation, with 0≤β≤1.0 for all signals. Probes with poor overall signals (p>0.05) were removed from analysis. Only probes positioned from −1,000 to+200 base pairs around transcription start sites (TSS) were analyzed. Heat maps were based on hierarchical clustering of β values using Euclidean distance and Ward’s algorithm, and all probes mapped to the genome (National Center for Biotechnology Information Build 36.3) using the bowtie algorithm and ultrafast and memory-efficient alignment of short DNA sequences (Genome Biology, 10, R25) with genome annotation matching the release of the Ensembl database. X-linked genes were removed from the analysis.

### Teratoma Assay

iPSCs were expanded to>80% confluency on 6-well plates. For each teratoma, cells from 6 wells were treated with trypsin, washed in PBS, and resuspended in 100 µl PBS. Mice (NOD/SCID) were injected subcutaneously with a mixture of 100 µl cells+100 µl hESC qualified Matrigel (BD Biosciences). The mice underwent necropsy when teratomas became evident (after 6–8 weeks). Tumors were excised and tissues stained with hematoxylin and eosin (H & E) to identify the various germ layers.

## Supporting Information

Figure S1 HMGA1 does not alter proliferation in hESCs. The MTT cell proliferation assay shows that the H9 hESCs transduced to express *HMGA1* grow at a similar rate to that observed in the control H9 hESCs, transduced with the GFP vector alone. This assay was done in triplicate; each time point shows the mean+/− the standard deviation.
(DOCX)

Figure S2 *HMGA1* promotes cellular reprogramming of IMR90. **A)** Reprogramming with HMGA1-OSKM results in more TRA-1-60+ iPSC colonies compared to controls. **B)** The HMGA1-OSKM TRA-1-60+ colonies are significantly larger than the control-OSKM colonies. Numbers represent µm diameters.
(DOCX)

Figure S3 Transgene expression in early stage reprogramming pools. Expression levels of the OCT4, SOX2, cMYC, and KLF4 transgenes were analyzed by qRT-PCR at day 12 and day 21 following the start of reprogramming in MSCs.
(DOCX)

Figure S4 HMGA1 KD targets pluripotency genes. Pluripotency genes (*SOX2, OCT4, cMYC, LIN28*) are repressed following knockdown of *HMGA1*, assessed 24 hours following siRNA transfection.
(DOCX)

Figure S5 Global promoter DNA methylation signatures in HMGA1-OSKM or control-OSKM iPSCs. Unsupervised hierarchical clustering of CpG loci shows the greatest variation across cell types. The 2D-hierachial cluster analysis, performed using the Euclidean distance on 38 cell lines, and 414 loci, places the cell lines described in this study into context in the complex network of methylation changes described in Ohm et al. [12]. The HMGA1-OSKM lines are marked in the top margin in blue, while the control-OSKM lines are marked in green. The partially reprogrammed cells collected at days 12 and 21 cluster on the right side with fibroblasts and other partially reprogrammed iPSCs, while the late passage HMGA1-OSKM or control-OSKM lines are found on the left with hESCs and other fully reprogrammed iPSC lines. Methylation patterns for most of the cancer cells (colon, breast, osteosarcoma, fibrosarcoma) located in the middle of the heat map are distinct from both the fibroblasts and pluripotent cells, with more extensive methylation globally and patterns that are negatively correlated with the methylation patterns observed in pluripotent cells. Dark blue – low methylation, light blue – high methylation.
(DOCX)

Table S1 Primers used in this study.
(DOCX)
