# Supplementary figures and images for: HMGA1 Reprograms Somatic Cells into Pluripotent Stem Cells by Inducing Stem Cell Transcriptional Networks
Source: PLoS One. 2012 Nov 15;7(11):e48533. doi: 10.1371/journal.pone.0048533 (PMC3499526; doi:10.1371/journal.pone.0048533)

**Supplementary Figure S1**


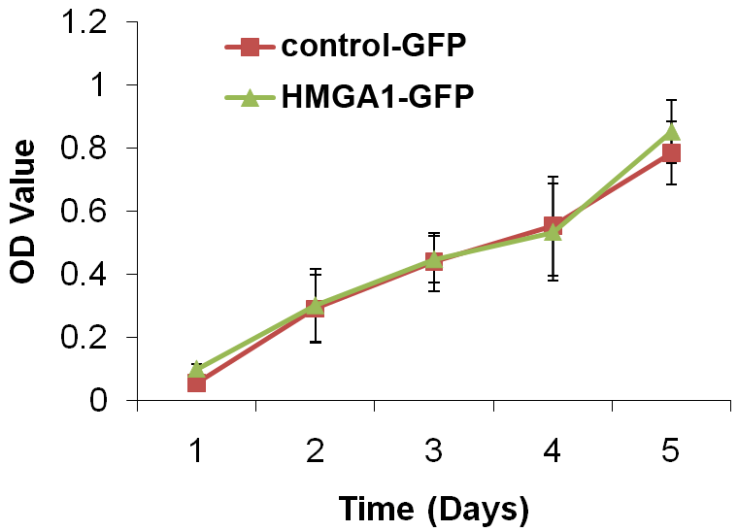

Supplement: Figure S1 — HMGA1 does not alter proliferation in hESCs. The MTT cell proliferation assay shows that the H9 hESCs transduced to express HMGA1 grow at a similar rate to that observed in the control H9 hESCs, transduced with the GFP vector alone. This assay was done in triplicate; each time point shows the mean+/− the standard deviation. (DOCX) [file pone.0048533.s001.docx]

**Supplementary Figure S2**


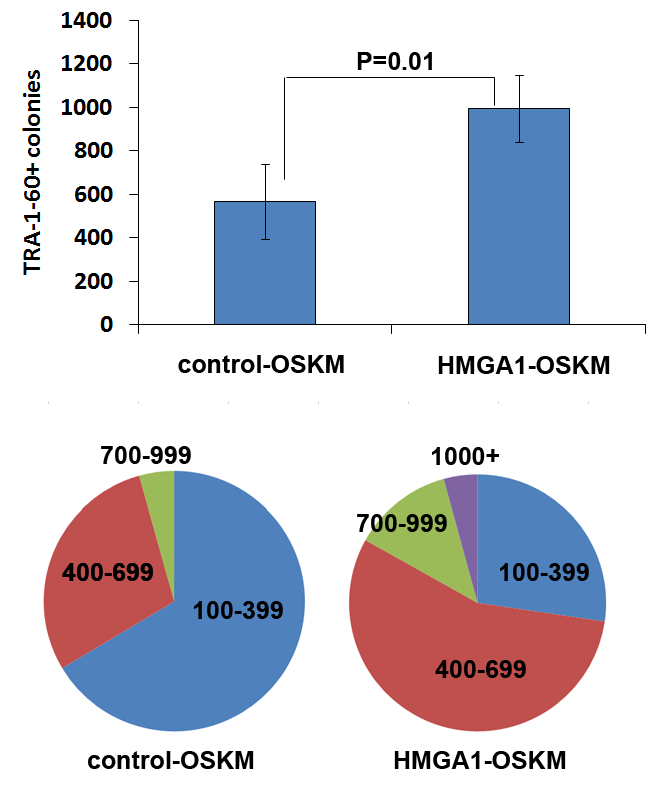

Supplement: Figure S2 — HMGA1 promotes cellular reprogramming of IMR90. A) Reprogramming with HMGA1-OSKM results in more TRA-1-60+ iPSC colonies compared to controls. B) The HMGA1-OSKM TRA-1-60+ colonies are significantly larger than the control-OSKM colonies. Numbers represent µm diameters. (DOCX) [file pone.0048533.s002.docx]

**Supplementary Figure S3**


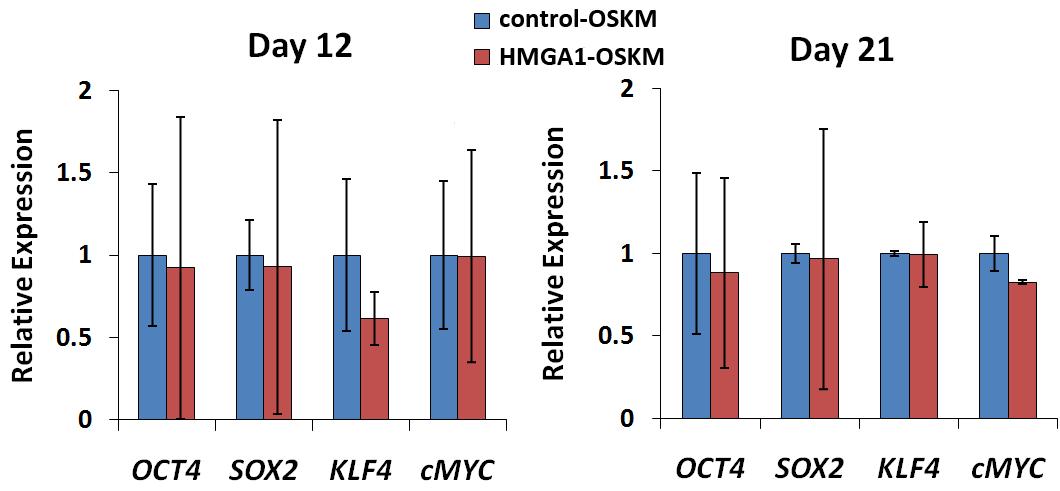

Supplement: Figure S3 — Transgene expression in early stage reprogramming pools. Expression levels of the OCT4, SOX2, cMYC, and KLF4 transgenes were analyzed by qRT-PCR at day 12 and day 21 following the start of reprogramming in MSCs. (DOCX) [file pone.0048533.s003.docx]

**Supplementary Figure S4**

**
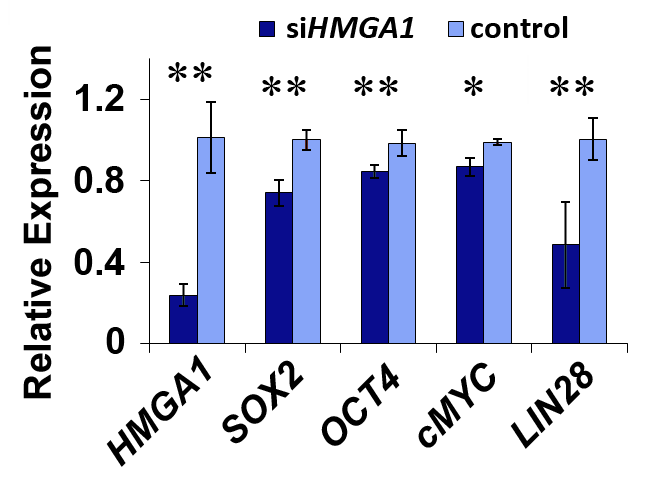
**

Supplement: Figure S4 — HMGA1 KD targets pluripotency genes. Pluripotency genes (SOX2, OCT4, cMYC, LIN28) are repressed following knockdown of HMGA1, assessed 24 hours following siRNA transfection. (DOCX) [file pone.0048533.s004.docx]

**Supplementary Figure S5**


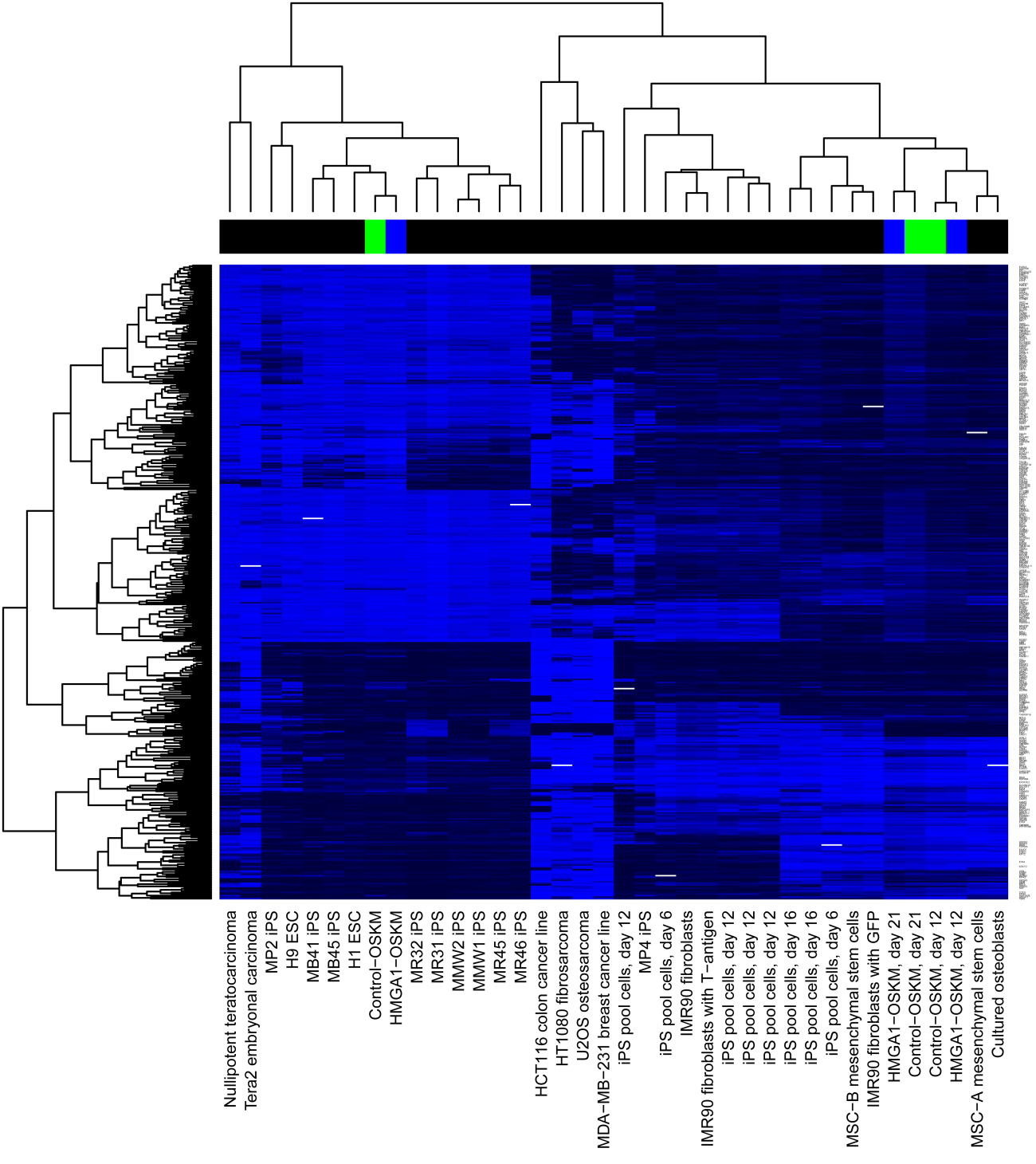

Supplement: Figure S5 — Global promoter DNA methylation signatures in HMGA1-OSKM or control-OSKM iPSCs. Unsupervised hierarchical clustering of CpG loci shows the greatest variation across cell types. The 2D-hierachial cluster analysis, performed using the Euclidean distance on 38 cell lines, and 414 loci, places the cell lines described in this study into context in the complex network of methylation changes described in Ohm et al. [12]. The HMGA1-OSKM lines are marked in the top margin in blue, while the control-OSKM lines are marked in green. The partially reprogrammed cells collected at days 12 and 21 cluster on the right side with fibroblasts and other partially reprogrammed iPSCs, while the late passage HMGA1-OSKM or control-OSKM lines are found on the left with hESCs and other fully reprogrammed iPSC lines. Methylation patterns for most of the cancer cells (colon, breast, osteosarcoma, fibrosarcoma) located in the middle of the heat map are distinct from both the fibroblasts and pluripotent cells, with more extensive methylation globally and patterns that are negatively correlated with the methylation patterns observed in pluripotent cells. Dark blue – low methylation, light blue – high methylation. (DOCX) [file pone.0048533.s005.docx]
