# Supplementary material for: HMGA1 Reprograms Somatic Cells into Pluripotent Stem Cells by Inducing Stem Cell Transcriptional Networks
Source: PLoS One. 2012 Nov 15;7(11):e48533. doi: 10.1371/journal.pone.0048533 (PMC3499526; doi:10.1371/journal.pone.0048533)
Supplement: Table S1 — Primers used in this study. (DOCX) [file pone.0048533.s006.docx]

**Supplementary Table S1: List of primers used in this study**

|  | | | |  |  |  |  |  |
| --- | --- | --- | --- | --- | --- | --- | --- | --- |
| **Gene** | **Forward Primer (5'-3')** | | |  | **Reverse Primer (5'-3')** | | |  |
| Endogenous |  |  |  |  |  |  |  |  |
| *SOX2* | CCC AGC AGA CTT CAC ATG T | | |  | CCT CCC ATT TCC CTC GTT TT | | |  |
| *cMYC* | TGC CTC AAA TTG GAC TTT GG | | |  | GAT TGA AAT TCT GTG TAA CTG C | | | |
| *OCT4* | CCC CAG GGC CCC ATT TTG GTA CC | | | | GGC ACA AAC TCC AGG TTT TC | | |  |
| *KLF4* | ACC CAC ACA GGT GAG AAA CCT T | | | | GTT GGG AAC TTG ACC ATG ATT G | | | |
| *LIN28* | CAA AAG GAA AGA GCA TGC AGA A | | | | ATG ATC TAG ACC TCC AGA GTT GTA GC | | | |
|  |  |  |  |  |  |  |  |  |
| *SOX1* | GAG ATT CAT CTC AGG ATT GAG ATT CTA | | | | GGC CTA CTG TAA TCT TTT CTC CAC T | | | |
| *Brachyury* | CAC CTG CAA ATC CTC ATC CTC AGT | | | | TGT CAT GGG ATT GCA GCA TGG A | | | |
| *Goosecoid* | CGC CTC GGC TAC AAC AAC TAC TTC TA | | | | ACG TTC ATG TAG GGC AGC ATC T | | | |
| *CXCR4* | CAC CGC ATC TGG AGA ACC A | | |  | GCC CAT TTC CTC GGT GTA GTT | | |  |
| *Sox17* | GGC GCA GCA GAA TCC AGA | | |  | CCA CGA CTT GCC CAG CAT | | |  |
| *hCG beta* | AGA GTG CAC ATT GAC AGC TGA G | | | | ATC ACC GTC AAC ACC ACC ATC TGT G | | | |
|  |  |  |  |  |  |  |  |  |
| Transgene |  |  |  |  |  |  |  |  |
| *Oct4* | TCT GGG CTC TCC CAT GCA TTC AAA | | | | AAC CTA CAG GTG GGG TCT TTC A ** |  |  |  |
| *Sox2* | GTG TGG TGG TAC GGG AAA TCA C ** | | | | TTC AGC TCC GTC TCC ATC ATG | | |  |
| *Klf4* | GTG TGG TGG TAC GGG AAA TCA C ** | | | | CGC GAA CGT GGA GAA GGA | | |  |
| *cMyc* | GTG TGG TGG TAC GGG AAA TCA C ** | | | | GTC ATA GTT CCT GTT GGT GAA GTT CA | | | |
| *HMGA1* | ACC ACA ACT CCA GGA AGG AAA CCA | | | | AAC CTA CAG GTG GGG TCT TTC A ** |  |  |  |

**located within the pMXs vector
